# Metabolic Syndrome: conceptual analysis in the nursing context

**DOI:** 10.1590/1518-8345.3008.3154

**Published:** 2019-08-19

**Authors:** Nuno Damácio de Carvalho Félix, Maria Miriam Lima da Nóbrega

**Affiliations:** 1 Universidade Federal da Paraíba, João Pessoa, PB, Brasil.; Universidade Federal do Recôncavo da Bahia, Centro de Ciências da Saúde, Santo Antônio de Jesus, BA, Brasil.; 2 Universidade Federal da Paraíba, Departamento de enfermagem em Saúde Coletiva, João Pessoa, PB, Brasil.

**Keywords:** Nursing, Concept Formation, Metabolic Syndrome, Risk Factors, Risk, Cardiovascular Nursing, Enfermagem, Formação de Conceito, Síndrome Metabólica, Fatores de Risco, Risco, Enfermagem Cardiovascular, Enfermería, Formación de Concepto, Síndrome Metabólico, Factores de Riesgo, Riesgo, Enfermería Cardiovascular, Autor correspondiente: Nuno Damácio de Carvalho Félix E-mail: nunof05@hotmail, com

## Abstract

**Objective:**

to analyze the metabolic syndrome concept and to identify its essential features, antecedents, and outcomes within the context of nursing.

**Method:**

conceptual analysis, based on the methodological steps of a model. We carried out an integrative review by accessing four databases online: *Medical Literature Analysis and Retrieval System Online, Scientific Electronic Library Online, Latin American and Caribbean Health Sciences Literature,* and Índice *Bibliográfico Español en Ciencias de la Salud*.

**Results:**

the essential features most frequently involved the diagnostic criteria of metabolic syndrome. Inadequate nutrition and physical inactivity were highlighted as the most common antecedents of the syndrome, and the outcomes were occurrences of cardiovascular disease and diabetes mellitus type 2. As implication, we highlight relevant empirical data to the broad definition of the concept.

**Conclusion:**

we could analyze the concept under study regarding essential features, antecedents, and outcomes, operationally defining it as a potential nursing phenomenon, which demands health care focusing on reducing risks and morbidity and mortality for cardiovascular diseases.

## Abstract (pt)

**Objetivo:**

analisar o conceito síndrome metabólica e identificar respectivos atributos essenciais, antecedentes e consequentes no contexto da enfermagem.

**Método:**

análise conceitual, a partir dos passos metodológicos de um modelo. Realizou-se revisão integrativa, por meio de acesso on-line a quatro bases de dados: *Medical Literature Analysis and Retrieval System Online, Scientific Electronic Library Online,* Literatura Latino-Americana em Ciências da Saúde e Índice Bibliográfico *Español en Ciencias de la Salud*.

**Resultados:**

os atributos essenciais mais frequentes envolveram os critérios diagnósticos da síndrome metabólica. Alimentação inadequada e sedentarismo destacaram-se como antecedentes mais comuns da síndrome, e os consequentes foram ocorrências de doenças cardiovasculares e diabetes mellitus tipo 2. Como implicação, tem-se o destaque de relevantes dados empíricos para definição ampla do conceito.

**Conclusão:**

foi possível analisar o conceito em estudo no que concerne aos atributos essenciais, antecedentes e consequentes, definindo-o operacionalmente como potencial fenômeno de enfermagem que demanda cuidados direcionados para redução do risco e da morbimortalidade por doenças cardiovasculares.

## Abstract (es)

**Objetivo:**

analizar el concepto síndrome metabólica e identificar respectivos atributos esenciales, antecedentes y consecuentes en el contexto de la enfermería.

**Método:**

análisis conceptual, desde los pasos metodológicos de un modelo. Se realizó la revisión integrativa, por medio de acceso online a cuatro bases de datos: *Medical Literature Analysis and Retrieval System Online, Scientific Electronic Library Online,* Literatura Latinoamericana en Ciencias de la Salud y Índice Bibliográfico Español en Ciencias de la Salud.

**Resultados:**

los atributos esenciales más frecuentes involucraron los criterios diagnósticos del síndrome metabólica. Alimentación inadecuada y sedentarismo se destacaron como antecedentes más comunes del síndrome, y los consecuentes fueron ocurrencias de enfermedades cardiovasculares y diabetes mellitus tipo 2. Como implicación, se tiene el destaque de relevantes datos empíricos para la definición amplia del concepto.

**Conclusión:**

ha sido posible analizar el concepto en estudio en el que concierne a los atributos esenciales, antecedentes y consecuentes, definiéndolo operacionalmente como potencial fenómeno de enfermería que demanda cuidados direccionados para la reducción del riesgo y de la morbimortalidad por enfermedades cardiovasculares.

## Introduction

The metabolic syndrome has drawn the attention of the scientific and professional community, not only due to the impact of each of their respective components, but mainly due to the high prevalence of cardiovascular risk factors^1^. According to a global estimate there is prevalence between 20 and 25% in the adult population^2^. In Brazil, the prevalence ranges around 9% in the adult and older-adult population, associated with the following variables: sociodemographic (age, education, marital status, and housing), behavioral (self-perception of health), and comorbidities (cerebrovascular accident, overweight, depression), in different ways between the sexes^3^.

This health condition has been widely studied by researchers, with varying criteria and existing definitions for diagnosis, particularities, and prevalence in population and age groups, aiming to discuss the accuracy of its assumptions^4-5^ and possible parameters that may be related such as neck circumference^6^ and presence of *Acanthosis nigricans* ^7^.

This syndrome was officially and primary described by Gerald Reaven in 1988, and was named “Syndrome X,” which comprised insulin resistance, hypertension, dyslipidemia, and diabetes mellitus, and did not include obesity, currently considered one of the basal pathogenic factors^4,8^. Other concepts used to characterize it are mentioned such as insulin resistance syndrome, new world syndrome, plurimetabolic syndrome, deadly quartet syndrome, and dyslipidemia syndrome^9^.

Likewise, concerning the definition, the metabolic syndrome concept, currently accepted, is nonuniform on health literature, whether national^10^ or international^11^, which evidences the understanding of disease or disorder, even though publications^1,12^ spread the understanding that it is a set of cardiovascular risk factors. Hence, we highlight a gap in the knowledge about the idea that a broader definition for the metabolic syndrome concept would favor the development of health care in practice, teaching, and research, through innovative approaches^13^, by inserting nursing care in such.

Within the context of nursing, risk factors that compose the syndrome refer to the field of performance of the discipline, such as the measurement of waist circumference, blood pressure, and evaluation of laboratory parameters^14^, in order to comprise a nursing phenomenon, for which research should be developed to introduce new and relevant knowledge such as the analysis of the concept and applicability as human responses to inadequate life habits. Therefore, our objective was to analyze the metabolic syndrome concept and to identify its essential features, antecedents, and outcomes within the context of nursing.

## Method

This is a conceptual analysis, based on the methodological steps of the Walker and Avant’s Model^15^, covering the process of clarification of meanings of terms and their respective definition, in such a way that researchers and readers share a common language, especially, when a concept requires “clarification,” as stated by the authors of the model, with improved definition for research, development of theories, or the clinical practice of nursing.

Six out of eight steps of the model were developed^15^: selection of the concept; delimitation of the analysis objectives; identification of the uses of the concept in the literature; determination of essential features; identification of antecedents and outcomes of the concept under analysis; and definition of empirical references of the studied concept. We considered these steps, since the study specifically involved the analysis and definition of the metabolic syndrome concept, which we could contemplate without the identification of a case-model and additional cases.

For the selection of the metabolic syndrome concept, we considered such identification in clinical practice and research, as a phenomenon of significant occurrence in people attended by nurses in the context of Primary Health Care. Moreover, since it is a condition that puts individuals at risk of complications, it demands deepening and clarifying the concept, the knowledge produced about the syndrome, and its insertion as a nursing phenomenon, and, thus, in line with the aforementioned model^15^.

This analysis demand is directly related to the purpose of our study, previously presented. In order to achieve it, we question: what is the reason for this concept analysis? Regarding the identification of possible uses of the concept, we searched the literature for synthesis and for understanding how this knowledge is focused or applied, implicitly and explicitly.

In our study, in which we analyzed the concept within the context of nursing, some criteria were deemed relevant for identifying uses in articles: being produced by nurses and or published in nursing journals or related areas; directly contemplating the syndrome, cardiovascular risk factors, and/or overweight and obesity in the content; clearly presenting the concept in the title and or in the development of the article; exposing data relevant to the analysis for composing the concept as a nursing phenomenon; and prioritizing articles with higher level of scientific evidence.

Concerning antecedents, features, and outcomes, the model^15^ define them, respectively, as: events that happen a priori to the phenomenon (required for its occurrence); words or expressions that repeatedly emerges in the literature, which demonstrates the essence of the concept; and events or situations that occur a posteriori to the phenomenon, respectively. We drew our attention to the exclusive criterion, according to which something cannot be, at the same time, a feature, an antecedent, and an outcome.

To follow this step, we conducted an integrative literature review, according to the development stages of this type of review in nursing^16^, because we understand the relevance of the systematic and operational approach of this process, in such a way we can achieve results according to our predetermined goal. In the productions selected for the review, we sought the use and definition of the concept, a *sine qua non* for inclusion in the study, besides frequency, adequacy, and direct correlation with the syndrome itself.

Articles were surveyed from a search conducted between March and April 2018, in the databases *Medical Literature Analysis and Retrieval System Online* (MEDLINE via Pubmed) and *Scientific Electronic Library Online* (SciELO), accessed through the Portal of Periodicals of the Coordination for the Improvement of Higher Education Personnel Foundation (*Coordenação de Aperfeiçoamento de Pessoal de Nível Superior* – CAPES) (CAPES), and the *Latin American and Caribbean Health Sciences Literature* (LILACS) and the Índice *Bibliográfico Español en Ciencias de la Salud* (IBECS), via Virtual Health Library (VHL). We used the operator “AND” and the following controlled and indexed keywords in the *Medical Subject Headings* (MeSH) and the *Health Sciences Descriptors* (DeCS), respectively: “Nursing,” “Metabolic Syndrome X,” “Risk Factors”; and the same words in Portuguese, “Enfermagem,” “Síndrome X Metabólica,” and “Fatores de risco,” in addition to the uncontrolled descriptor “síndrome metabólica,” [metabolic syndrome] at the time not updated in DeCS.

**Figure 1:**
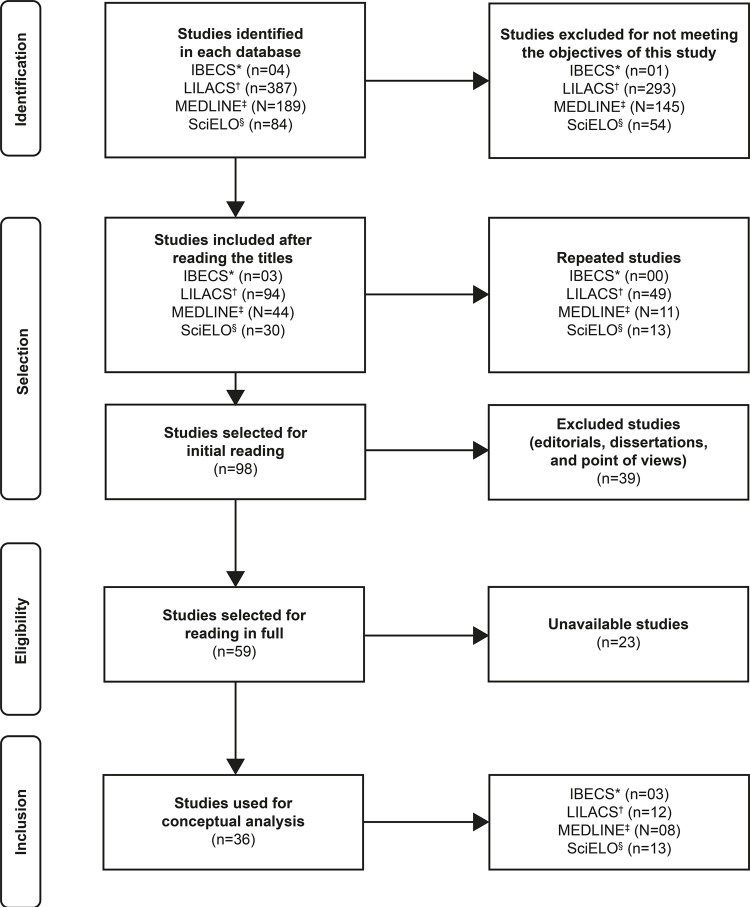
– Flowchart of search in the databases. João Pessoa, PB, Brazil, 2018 Note: *IBECS – Índice *Bibliográfico Español en Ciencias de la Salud;* †LILACS – *Latin American and Caribbean Health Sciences Literature*; ‡MEDLINE – *Medical Literature Analysis and Retrieval System Online*; §SciELO *– Scientific Electronic Library Online*

Source: Adapted^(17)^.

In this selection, we established the inclusion criteria: full papers electronically available, in English, Portuguese, or Spanish, indexed in the last ten years, on human beings, regardless of age, population group, or correlation with diseases. We excluded from the studies repeated and editorial articles, dissertations, theses, point of views, and case studies. These criteria, in addition to the methodological strictness, were paramount to reduce biases of the study. For the search process and selection of the articles, specifically, we followed the recommendations of the *Preferred Reporting Items for Systematic Reviews and Meta Analyses* (PRISMA)^17^guide, which are detailed in Figure 1.

After the search procedure, we read the titles and abstracts of the selected articles to verify if they met the established inclusion criteria and, later, to read them in full. We used the PICO strategy, representing the acronym for “patients,” “intervention,” “comparison,” and “outcomes”, key elements of the research questions, namely: P – individuals with metabolic syndrome; I – does not apply; C – does not apply; and O – essential features, antecedents, and outcomes.

Extraction of empirical data was performed by the following questions: how authors define the metabolic syndrome concept? Which habits, behaviors, events, situations, and phenomena contribute to the development of the syndrome? What features and peculiarities were mentioned by the authors? What are the consequences of the diagnosis and non-follow-up of the syndrome?

For data collection, we used an instrument previously prepared with the characterization of literature (author, year, database, type of study, and title) and the empirical data of the selected articles (definition, concept, antecedents, essential features, and outcomes). Then, data were coded and distributed into categories and subcategories, whose “essential features” of the syndrome were composed of characteristics related to the contexts of the nursing practice; the “antecedents” and “outcomes” categories were organized into subcategories according to the potential of change or not, and the temporal aspect (short, medium, and long term), based on critical analysis, respectively.

We drew our attention to the direct relationship between the elements and the studied phenomenon, in an analytical, manual, thorough, and exhaustive way of the selected articles, classifying them according to the level of evidence^18^: evidence from systematic review or meta-analysis of relevant, randomized and controlled clinical trials, or from guidelines based on systematic reviews of clinical trials with randomization, controlled (Level I); evidence from at least one clinical trial with randomization, controlled and well-delineated (Level II); evidence from well-defined, controlled studies without randomization (Level III); evidence from cohort studies or case-control studies (Level IV); evidence from systematic review of qualitative and descriptive studies (Level V); evidence from single study, descriptive or qualitative (Level VI); evidence from opinion of authorities or reports of expert committees (Level VII).

Finally, empirical references were gathered to create a definition, understood as categories or classes of observable phenomena that demonstrate the occurrence of the concept by an operational definition of such^15^. Results were critically analyzed and arranged in a table, with absolute and relative frequencies of features, antecedents, and outcomes in relation to the number of publications, in addition to figures. Then, we discussed the state of the art produced in relation to the concept under study and the critical analysis of the evidenced data, in addition to their correlation with nursing.

Our study did not demanded submission to the Research Ethics Committee, since we used literature to analyze the concept, not involving humans, directly or indirectly.

## Results

**Figure 2:**
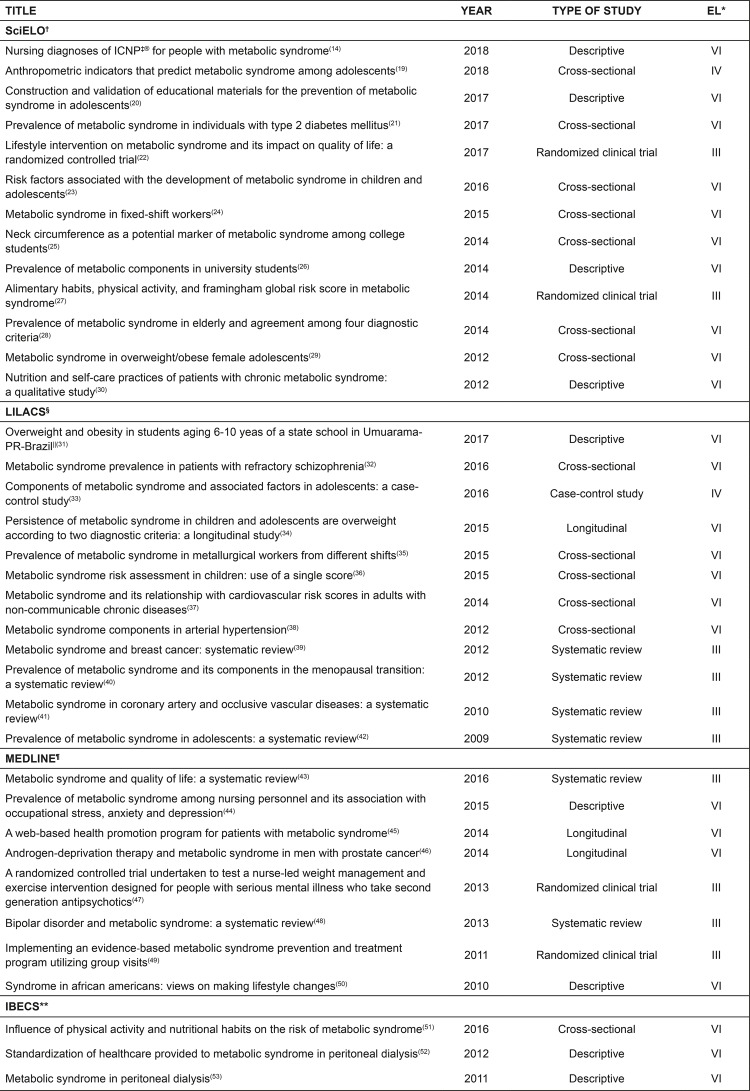
– Classification of articles included in the analysis of the metabolic syndrome concept. João Pessoa, PB, Brazil, 2018 Note: *EL – Evidence level; †SciELO *– Scientific Electronic Library Online*; ‡ICNP^®^ – International Classification for Nursing Practice; §LILACS – Latin American and Caribbean Health Sciences Literature; ||PR – Paraná state, Brazil; ^¶^MEDLINE – *Medical Literature Analysis and Retrieval System Online*; **IBECS – Índice *Bibliográfico Español en Ciencias de la Salud*

We selected 36 articles produced and/or published by nurses in nursing journals or related areas. These articles mostly involved cross-sectional (n=13) and descriptive (n=9) studies, with Level VI classification of scientific evidence, depicting the profile of nursing productions about the concept under study. In Figure 2 we present the profile of the selected articles, according to title, author, year of publication, type of study, and level of evidence.

**Figure 3:**
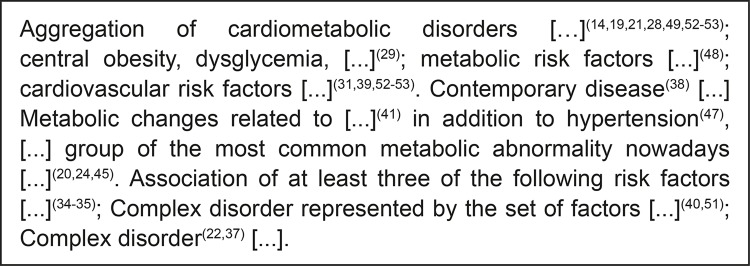
– Examples of expressions used by the authors to define the metabolic syndrome concept. João Pessoa, PB, Brazil, 2018

Publications that prevailed were those indexed in the database SciELO (36.1%), published in Brazil (75%), mostly (19.4%) in the year 2014. Through the critical analysis of the definitions of the concept in the publications, we observed that the expression “aggregation” was the most frequent initial term in definitions and, in our study, we used it to initially relate the essential features to the concept, antecedents, and outcomes. However, part of the studies conducted by the nursing field still defines the syndrome as a condition of disease, disorder, or abnormality, as shown in Figure 3.

In Table 1 we present the absolute and relative frequencies of the concepts and factors related to antecedents and outcomes of the analyzed concept, in which we verified frequency of diagnostic characteristics of the syndrome as essential features in 100% of the publications, as well as in antecedents and outcomes related to inadequate nutrition and physical inactivity, and the occurrence of cardiovascular diseases and type 2 diabetes, respectively.

**Table 1:** – Frequency of features, antecedents, and outcomes of the metabolic syndrome concept, according to number of analyzed studies. João Pessoa, PB, Brazil, 2018

Concepts/related factors	*F	^†^%
**Essential features**		
High blood pressure	36	100
High fasting glucose	36	100
High triglycerides	36	100
High waist circumference	36	100
Low high-density lipoprotein cholesterol	36	100
Aggregation	07	19.4
Asymptomatic inflammation	06	16.7
Significant cardiovascular risk markers	03	8.3
Multifactorial etiology	02	5.6
Vulnerability	02	5.6
Demand for multidisciplinary approach	01	2.8
**Antecedents**		
Sedentary lifestyle	36	100
Inadequate nutrition	36	100
Unfavorable socioeconomic and educational condition	13	36.1
Smoking and alcoholism	10	27.8
Prevalence among different sexes, ethnicities, ages, and races	08	22.2
Genetic predisposition to cardiometabolic changes	08	22.2
Depression and anxiety	08	22.2
Inadequate working organization and conditions	07	19.4
Weight gain	07	19.4
Stress	07	19.4
Lack of knowledge	06	16.7
Deficit in self-care	06	16.7
High estrogen/progesterone and menopause	06	16.7
Use of psychotropic medication and polypharmacy	05	13.9
Impaired sleep and rest	04	11.1
Low adhesion	04	11.1
Family history of cardiovascular diseases	03	8.3
Feelings of frustration, sadness, failure, and guilt	02	5.6
Issues in working relationships	02	5.6
Lack of family and social support	02	5.6
Difficulty in interpersonal relationships	02	5.6
Dialysis	02	5.6
Bipolar disorder and schizophrenia	02	5.6
Hormone replacement or deprivation therapy	02	5.6
**Outcomes**		
Occurrence of cardiovascular diseases and diabetes mellitus type 2	36	100
Decreased life expectancy and premature morbidity and mortality	18	50
Impairment of quality of life	09	25
Risk of cardiovascular and cerebrovascular complications	09	25
Emotional impacts	06	16.7
Kidney diseases	04	11.1
High treatment costs and number of hospitalizations	03	8.3
Impacts on work performance and occupational diseases	03	8.3
Social isolation and risk of suicide	03	8.3
Neoplasms	02	5.6
Low self-esteem and negative self-image	02	5.6

Note: *F – Absolute frequency; †% – Relative frequency

**Figure 4:**
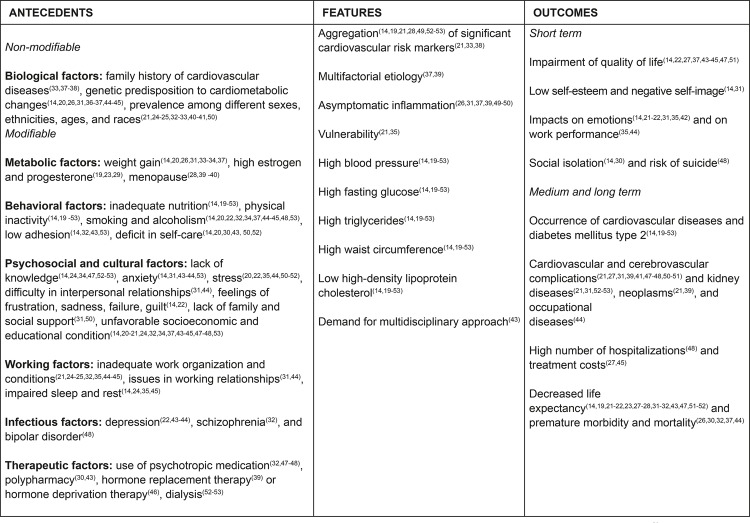
– Essential features, antecedents, and outcomes of the metabolic syndrome concept. João Pessoa, PB, Brazil, 2018

Through the results, we verified that the “metabolic syndrome,” as an objective phenomenon, features empirical indicators related to essential features, antecedents, and outcomes in the short, medium, and long term, to be clearly evidenced in clinical practice of nursing professionals, as we show in Figure 4.

The analysis provided structuring the broader and more comprehensive operational definition of the metabolic syndrome phenomenon, which is characterized by the aggregation of significant cardiovascular risk markers of multifactorial etiology, related to asymptomatic inflammation that predisposes the individual to vulnerability. It involves the identification of at least three diagnostic criteria, such as high waist circumference, high fasting glucose, high blood pressure, high triglycerides, and/or low high-density lipoprotein cholesterol, according to the adopted parameter and the demand for multidisciplinary approach within the nursing context.

## Discussion

Regarding definitions that we found in the studies, we observed prevalence of the use of expressions that relate the metabolic syndrome to a pathological condition, which is common, but inadequate. This fact is related to the remnants of the culture of the biological health, focused on disease, still evident in the literature. Our study contributes to clarifying and advancing the metabolic syndrome concept, defining it by a more comprehensive point of view and based on theoretical references for the use on the part of nursing professionals and scientific community and other health fields.

The “Antecedents” category was organized into three categories and eight subcategories, with syndrome-related factors evidenced in literature and analyzed concerning its insertion in the field of nursing. Regarding biological factors, categorized in our study as “non-modifiable,” we found prevalence of the phenomenon from varying diagnostic criteria, in different ethnicities, races, ages, and both sexes, in particular the high prevalence among children^36^, adolescents^23,33^, and young adults and older adults^32^, and these groups of individuals are constantly attended by the nursing staff in programs of the Primary Health Care.

These factors can vary and be aggravated when related to family history of cardiovascular disease^33,37 -38^, in addition to genetic predisposition to cardiometabolic changes, widely described in the literature^19-20,26-27,31,33,35,37-39,49^. However, longitudinal and experimental studies are still incipient, studies which should focus on the understanding on the relationship between these factors and the phenomenon, in which nursing is inserted in this collaboration to the progress of science on the subject.

Results related to modifiable factors were widely discussed. Metabolic factors featured alarming data on the Brazilian adult population, according to which more than 18.9% of Brazilians are obese, regardless of sex^54^. The youth population was highlighted, in which there is a high prevalence of overweight and changes in the lipid profile of these individuals. These factors may pose risk for further metabolic syndrome and cardiovascular diseases^26^, which require nursing interventions to reduce them.

Therefore, the development of effective health care and the encouragement to public policies are suggested, policies which should act towards the awareness of a healthy lifestyle, focusing on the family, prioritizing older people without, however, disregarding younger people^28,32-33^. This comprises the nursing care that must be systematically provided and registered according to a standardized language, from a classification system.

For the older population, menopause transition promotes increase in the measurement of waist circumference, blood pressure, fasting blood glucose, triglycerides, and decrease in the high-density lipoprotein cholesterol, more expressive in the first two^40^. Hence, regarding the relation between the syndrome, menopause transition, and age, most studies showed that menopause transition was an independent predictor^29,40^; however, there is need for studies with more robust designs to establish the relation of cause and effect.

Concerning behavioral factors, we identified more studies with high level of scientific evidence, especially publications in the nursing field and/or developed by nurses. Authors of national^14^ and international^5^ studies point that nurses need to consider such factors when planning and prescribing nursing interventions directed to this clinical profile and population, in such a way to achieve results relevant to the nursing practice.

According to results of another study^27^, nurses’ recommendation on nutrition and physical activity, in all groups, may be deemed a paramount tool for the overall treatment of patients with metabolic syndrome, since there were positive results concerning metabolic and cardiovascular parameters, with encouragement for changing lifestyle to improve the quality of life of this population.

Studies on several types of nursing interventions^22,43,47,51-52^, healthcare promotion programs, Web-based^45^, of visits^49^, and self-care^30^ to changes in lifestyle and increased adherence among patients with the syndrome highlight impacts on the reduction of metabolic parameters and improvement in the quality of life, with beneficial effects on metabolic parameters, in particular, in weight loss and waist circumference.

As for the most frequent antecedents, their set negatively acts on the lipid profile and increases the prevalence of the syndrome and, therefore, the risk of cardiovascular disease^22,27,30,51^. Moreover, attention is draw to the effective development of healthcare actions, based on knowledge and adherence to preventive behaviors for decreasing diseases resulting from the syndrome^33^. Nurses are members of the participatory process of identifying these human responses and providing guidance for improving life habits and adherence to measures of healthcare promotion.

Concerning psychosocial and cultural factors, the literature^44^ highlights the correlation between metabolic syndrome variables and anxiety (p=0.022), and between the syndrome and stress (p=0.008). Nursing staff must seek social and family support, in such a way it may help in identifying ways to communicate with professionals^50^. In this situation, it is important to develop environments for nursing interaction, with families and community, for a healthy nutrition routine, practice of physical exercise, and weight and stress control^20^.

As for working factors, the syndrome can be related to some variables, such as work, poor sleep quality, poor diet, sedentary lifestyle, alcoholism, smoking, absenteeism, and dissatisfaction with the work^24,35^. Concerning such factors, studies have been developed with nurses for understanding this phenomenon among these professionals, who will be attended by other nursing professionals, and that the phenomenon also occurs in this category, which needs to be recognized and monitored.

Infectious factors identified as antecedents to the syndrome, such as depression^22,43-44^, schizophrenia^32^, and bipolar disorder^48^, were found due to the number of hospitalizations and used drugs, adverse effects, and inadequate nutrition, correlated with therapeutic factors, in which nursing professionals participate in the process of monitoring and managing the care provided to these individuals.

In the category of Essential Features, clinical and biochemical indicators have been recurrent and are essential for the clinical management of the syndrome, but other relevant features were more clearly perceived to define and broadly understand this concept. Thus, more important than add or modify indicators, we must be able to identify them in the clinical practice, to invest in health education and prevention measures, and to encourage good life habits, with the exchange of experiences and adoption of good health practices^19^. These actions can be effectively carried out by nurses when they understand the magnitude of the phenomenon under their care and seek to improve knowledge, whether by searching evidence-based interventions or by researching to identify the best practice in scientific basis within several disciplines.

Hence, a multidisciplinary approach in health is necessary^22^ in order to reduce factors responsible for the emergence of the syndrome and its respective outcomes. Nurses routinely deal with the imbalance of these factors and have an important role on the diagnosis, healthcare planning, strategy interventions, and control of this syndrome^21^.

Nursing professionals use knowledge from other fields in their professional practice and efficiently implement them, since they monitor, in a more longitudinal way, people affected by this syndrome. These professionals, within a multidisciplinary work, must know the concept of the phenomenon and engage in developing actions that promote cardiovascular health in people affected with the syndrome and which reduce the consequent morbidity and mortality.

The categorized features enabled the creation of an operational definition for the metabolic syndrome concept as a nursing phenomenon, in order to be used in the planning and implementation of nursing care and in the field of teaching and research, in such a way to reduce the antecedents and the respective outcomes of short, medium, and long term – and in these last we evidenced high frequency of the outcome related to the occurrence of cardiovascular disease and diabetes mellitus type 2^14,19-53^. We understand that such outcome comprises a relevant susceptibility to prevention, when performed effectively and comprehensively, being inserted into nursing in primary-level services, through evidence-based care, aiming at reducing the burden of the disease.

Hence, empirical data we presented in this study involve the nursing context, whereas the metabolic syndrome phenomenon comprises a set of features with antecedents and outcomes which are sensitive and verifiable in the working process within the area, actively inserted in the development of technologies for the prevention of metabolic syndrome^20^, establishing diagnoses, outcomes, and nursing interventions to the theoretical-practical improvement in nursing.

Among the limitations, we found not using all the steps proposed by the theoretical reference as well as not carrying out the analysis by experts. Such did not impair the scope of the objective, since we sought to critically analyze the evidenced empirical data, in addition to the methodological strictness. Further research should enhance the development of the concept, filling in the existing gaps, through studies with experimental designs to advance in the knowledge about the phenomenon and the application of the concept in the professional practice.

As a contribution, we mention, in particular, the introduction of scientific evidences to the understanding and discussion on metabolic syndrome as a nursing phenomenon, to the extent it collaborates to advances in the theoretical knowledge within the field of health, due to the prominence of new and relevant empirical data, such as the involved psychosocial and cultural factors and the prospect of vulnerability in which people with the syndrome are inserted as well as the broadening of a concept relevant to health policies and healthcare programs directed at healthcare promotion and cardiovascular prevention, with the active participation of nursing.

## Conclusion

The results of the analysis of the metabolic syndrome concept allowed the identification and articulation of the essential features of the phenomenon in the context of nursing, in addition to its most frequent antecedents and outcomes such as diagnostic criteria, life habits, and clinical impairment due to cardiometabolic diseases, corroborating the overall literature on the subject.

Our analysis of empirical data provided structuring the broader and more comprehensive operational definition of the metabolic syndrome concept, which is characterized by the aggregation of significant cardiovascular risk markers of multifactorial etiology, related to asymptomatic inflammation that predisposes the individual to vulnerability. It involves the identification of at least three diagnostic criteria, such as high waist circumference, high fasting glucose, high blood pressure, high triglycerides, and/or low high-density lipoprotein cholesterol, according to the adopted parameter and the demand for multidisciplinary approach within the nursing context.

Understanding the scope of the concept under analysis is required to its use in the practice, teaching, and research in nursing and health, and it enables the recognition of variables involved in this phenomenon to guide nurses and nursing staff in the process for identifying human responses of individuals affected with the syndrome and for planning the nursing cardiovascular care.

These professionals should engage in the improvement of the concept and in the verification of the emergence of this syndrome, in such a way to contribute to the development of cardiovascular prevention within these patients and broaden research in the metabolic syndrome as a nursing phenomenon, through existing and developing theoretical references.
